# Effect of Residential Greenness and Nearby Parks on Respiratory and Allergic Diseases among Middle School Adolescents in a Chinese City

**DOI:** 10.3390/ijerph16060991

**Published:** 2019-03-19

**Authors:** Linyan Li, Jaime E. Hart, Brent A. Coull, Shi-jie Cao, John D. Spengler, Gary Adamkiewicz

**Affiliations:** 1 Department of Environmental Health, Harvard T.H. Chan School of Public Health, Boston, MA 02215, USA; jaime.hart@channing.harvard.edu (J.E.H.); spengler@hsph.harvard.edu (J.D.S.); gadamkie@hsph.harvard.edu (G.A.); 2 Channing Division of Network Medicine, Department of Medicine, Brigham and Women’s Hospital and Harvard Medical School, Boston, MA 02215, USA; 3 Department of Biostatistics, Harvard School T.H. Chan School of Public Health, Boston, MA 02115, USA; bacoull@gmail.com; 4 Academy of Building Energy Efficiency, School of Civil Engineering, Guangzhou University, Guangzhou 510006, China; shijie.cao@gzhu.edu.cn

**Keywords:** residential greenness, NDVI, distance to park, asthma, pneumonia, rhinitis, eczema

## Abstract

Research on the health impacts of green environments has mainly been conducted in developed countries. Differences in the urban forms between China and Western countries make it essential to understand the role of greenspace in Chinese settings. From 2014 to 2015, middle school students (*n* = 5643) in Suzhou, China were enrolled in a study on the health effect of residential greenness. The normalized difference vegetation index (NDVI) and distance to the nearest park were calculated for each home address. Logistic regression was performed to test associations between exposure and self-reported doctor diagnoses of asthma, pneumonia, rhinitis, and eczema, adjusting for important confounders. No statistically significant associations were observed for any seasonal NDVI-based measures. However, the proximity of the participants’ residences to the closest park showed an inverse relationship to reported symptoms. The odds ratios for the furthest quartile compared to the closest quartile based on the distance to the nearest park were 0.58 (95% CI: 0.35, 0.99), 0.70 (95% CI: 0.50, 0.96), 0.92 (95% CI: 0.74, 1.15), 0.97 (95% CI: 0.76, 1.24), 0.86 (95% CI: 0.68, 1.10) for current asthma, ever asthma, ever pneumonia, ever rhinitis, and ever eczema, respectively. These findings focused on a single Chinese city and suggest that exposure to natural vegetation in urban areas may affect health through various pathways.

## 1. Introduction

As a component of infrastructure in modern cities, public greenspace can offer opportunities for recreational and social interactions. Additionally, these public places might provide other important benefits such as promoting physical activity, mitigating noise and air pollution, storm water management, enhancement of biodiversity, and stress reduction [1,2,3,4]. 

Several studies have examined the relationship between greenspace and atopic outcomes, but there were mixed findings. Some studies posit greenspace as a potential source for allergens and respiratory irritants [5]. Pilat et al. [6] found no statistically significant results for normalized difference vegetation index (NDVI), canopy cover, and asthma. Dadvand et al. [7] found living close to forests was positively associated with current allergic rhinoconjunctivitis, and proximity to parks was positively associated with current asthma and current allergic rhinoconjunctivitis. Fuertes et al. [8] studied the association between greenspace and allergies in two German areas, and found positive associations in the urban area but negative associations in the rural area. Another study by Fuertes et al. [9] tested the association between residential NDVI and allergic disease and found different trends within seven birth cohorts based in Sweden (BAMSE), Australia (MACS), Netherland (PIAMA), Canada (CAPPS and SAGE), and Germany (GINIplus and LISAplus). On the other hand, the biodiversity created by greenspace can be protective against inflammatory conditions, known as the “biodiversity hypothesis”, and has been supported in several studies [10,11,12]. Recently, Tischer et al. [13] examined the effect of greenness on children’s respiratory health and found proximity to residential greenness to be protective of bronchitis in the Mediterranean region and protective of wheezing for children in the Euro-Siberian region. James et al. [14] reviewed the health benefits of greenness and found while greenness is protective against adverse mental health outcomes, cardiovascular disease, and mortality, the health benefits are less conclusive for birth outcomes and respiratory disease. Because of these contradictory findings, it is important to add evidence to our understanding of the mechanisms by which greenspace affects respiratory health, especially in regions where such studies have not been conducted. To date, most studies on greenspace have been conducted in developed Western countries. James et al. [14] recommended studies on the impacts of greenspace on health in developing countries, especially in Asia. The modern form of Chinese cities is characterized by high residential density and intensively mixed land-use [15], which is a significant difference from Western countries. The difference in urban forms between China and Western countries related to ecological amenities has only recently been recognized as an important element [16,17,18]. National policy is now encouraging inclusion of more greenspace in urban development [19]. Therefore, understanding the relationship between greenspace in urban areas and health will be immediately relevant to urban planning in China. The objective of our study was to explore the relationship between greenspace (measured using both NDVI and nearby parks) and a wide range of respiratory and allergic health outcomes in children in a Chinese city. The goal was to provide evidence for policy making and public education. 

## 2. Methods

### 2.1. Study Population

From October 2014 to January 2015, we conducted a cross-sectional survey in Suzhou, China. As part of a larger study of environmental impacts on health, we randomly selected 4 schools within each district (six districts in total, with a total of 140 schools) and finally recruited 12 middle schools to represent different urban forms in Suzhou. Between October 2014 and January 2015, over 6000 middle school students and their parents/guardians received questionnaires from their teachers and were asked to return them within one week. A total of 5891 valid questionnaires were returned, for a response rate of 82.9%. The study protocol was approved by the Harvard T.H. Chan School of Public Health human subjects committee and local ethics committees. The adolescents’ parents or trustees provided written informed consent.

### 2.2. Exposure Assessment

Surrounding NDVI and distance to the nearest park were used to assess exposure to natural vegetation for our study population. Data on NDVI were captured from the National Aeronautics and Space Administration (NASA) Landsat satellite imagery of the Earth’s surface. Calculations of NDVI utilize the ratio between visible and near-infrared light reflected by vegetative growth and is expressed as a vegetation density from −1 to +1 [20]. Suzhou has four distinct seasons. Therefore, we used the most cloud-free NDVI images (cloud cover rate < 10% for all images) within a season from Satellite Landsat 8 (grid size: 30 m) to quantify the greenspace exposure in the spring (16 March), summer (22 June), fall (26 October), and winter (29 December) seasons. Average NDVI was calculated to represent the annual 2014 residential greenspace exposure to correspond to the time period of questionnaire administration. As the specific spatial scale at which greenspace may impact each of our selected outcomes was not known, we chose a range of commonly examined buffer sizes around each participants’ home addresses (100 m, 200 m, 500 m, and 1000 m) and calculated the average NDVI values within each buffer size [4,21,22,23,24]. Geographic Information System (GIS)-calculated distance from participants’ home to the nearest park was used as another exposure variable [4,7], and was categorized based on quartiles. Home addresses were geocoded using manual searches in Google Maps to obtain their coordinates. The positional error was on average 5.4 m, identified as the best performing georeferencing tool by Ribeiro et al. [25]. The match rate was 95.9%. We obtained maps of Suzhou city from Baidu Map (Similar to Google Maps, but a Chinese version), which contained 260 parks. These included greenspaces for recreational purposes, and exclusive sporting facilities were not included. The list of parks obtained from Baidu Map is provided in Table S1. The coordinates of the parks are for their street addresses, which can be interpreted as the entrance to the parks. Euclidean distance between each residence and the nearest park was calculated using ArcMap version 10.2.2 (ESRI, Redlands, CA).

Several confounders were included in our analyses. Questions about the children’s age, sex, family history of asthma, and environmental tobacco smoke at home were asked in the questionnaire with binary options. The father’s level of education was obtained by asking the participants to choose from four different options: primary school, middle school, high school, and college and above. We also obtained information on PM2.5 concentrations near each of the home addresses. There were 24 air monitoring stations in Suzhou that had data on PM2.5. We calculated the average value of PM2.5 for each monitor during the study period and interpolated to calculate the concentration at each home address. Dampness, mold, and pet information were obtained by asking binary questions on whether or not the child’s room had dampness or mold problems and whether there was a pet at home.

### 2.3. Outcome Assessment

Questions on diagnosis of asthma, pneumonia, rhinitis, and eczema were based on the China, Children, Home and Health study [26], which used questions adapted from the previously validated International Study of Asthma and Allergies in Childhood (ISAAC) questionnaire [27]. “Doctor-diagnosed asthma”, “doctor-diagnosed pneumonia”, “doctor-diagnosed rhinitis”, and “doctor-diagnosed eczema” were determined by positive answers to the questions: “Has the child ever been diagnosed with asthma (pneumonia, allergic rhinitis, eczema) by a doctor? (Yes/No)”, respectively. Current asthma was defined as the child ever having had doctor-diagnosed asthma and wheezing symptoms in the past 12 months [7].

### 2.4. Statistical Analysis

Multilevel logistic regression models were performed to test the associations between our exposure measures and each of the outcomes identified above. Crude models adjusted for adolescents’ age and sex, and a priori fully-adjusted models were further adjusted for environmental tobacco smoke (ETS) at home, parental education, and parental history of asthma. The confounding effects of air pollution, pets in the home, and dampness and mold were tested by adding these variables to the fully-adjusted model. Results were presented as odds ratios (ORs) and 95% confidence intervals (CIs) for an interquartile range (IQR) increase in annual NDVI values after assessing deviations from linearity using splines. To identify whether there was any seasonal effect, NDVI in four seasons were also separately examined. For distance to a park, ORs were presented compared to the quartile of participants living closest to a park. Effect modification by sex and socioeconomic status (SES, which in our study is represented by parental education) was tested by adding multiplicative interaction terms to the models, as well as by stratification. The potential clustering of response by schools was accounted for by regressing a multi-level model with a random intercept for schools. This random-school effect is included in all statistical models. *P*-values less than 0.05 were considered statistically significant. All computations were carried out using R version 3.2.3, and “glmer” in the “lme4” package was used for multilevel regression.

## 3. Results

A total of 5891 middle school adolescents and their parents/guardians participated in our study. Of these, 5643 participants provided their home addresses. There was no statistically significant difference in outcome prevalence or key covariates between participants who provided residential addresses and those who did not. Demographic features of the participants included in the analyses are shown in Table 1. A majority of adolescents (51.6%) were male and most were between 12 and 15 years old. The prevalence of doctor-diagnosed asthma, pneumonia, rhinitis, and eczema were 9.8%, 20.7%, 20.2%, and 18.5%, respectively.

**Figure 1:**
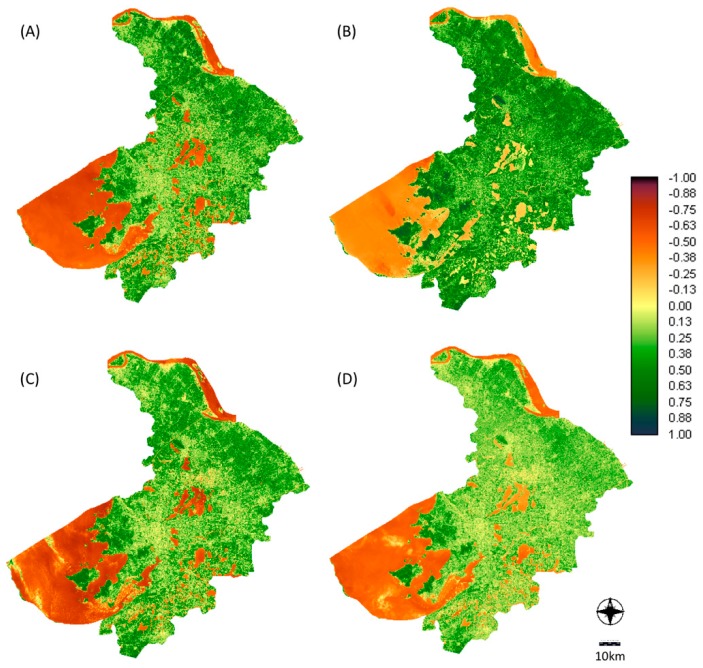
Distribution of NDVI in Suzhou, China 2014: (**A**) 16 March; (**B**) 22 June; (**C**) 26 October; (**D**) 29 December.

Figure 1 shows the spatial patters of the seasonal NDVI measures. Overall and season-specific median NDVI values are shown in Table 2. The median NDVI values within the different buffer areas did not vary substantially. The median NDVI value was highest in the summer, followed by spring, and lowest in fall and winter. For distance from home to the nearest park, the median distance in our study was 903 m, with the first quartile at 600 m and third quartile at 1348 m.

Table 3 shows the odds ratios from the crude and fully-adjusted multi-level logistic regression models for associations between interquartile increases in residential annual average NDVI values and health outcomes. As stated in the methods section, the fully-adjusted model included child’s age and sex, ETS at home, parental education, and parental history of asthma. Maternal smoking may be an important confounder that was not on our list, as this is very rare in the Chinese population, as reflected in the dataset we collected, and we thus we did not have enough information to analyze it. Results for the age and sex adjusted model and fully-adjusted model were similar. There were no statistically significant associations observed for residential NDVI values and any of the examined outcomes. Similar patterns were observed in models using seasonal measures of NDVI, and sex did not appear to modify these associations (data not shown).

Table 4 presents the crude and fully-adjusted odds ratios assessing distance to the nearest park. The ORs for people in the quartile living farthest from parks compared to those living closest to parks were 0.58 (95% CI: 0.35, 0.99), 0.70 (95% CI: 0.50, 0.96), 0.92 (95% CI: 0.74, 1.15), 0.97 (95% CI: 0.76, 1.24), 0.86 (95% CI: 0.68, 1.10) for current asthma, ever asthma, ever pneumonia, ever rhinitis and ever eczema, respectively. There were no statistically significant interaction terms between any outcome and gender or parental education. The *p*-values for the interaction term between gender and categorical distance to a park were 0.86, 0.87, 0.72, 0.81, and 0.11 for current asthma, ever asthma, ever pneumonia, ever rhinitis, and ever eczema, respectively. The *p*-values for the interaction term between the father’s education and categorical distance to a park were 0.87, 0.37, 0.39, 0.88, and 0.73 for current asthma, ever asthma, ever pneumonia, ever rhinitis, and ever eczema, respectively. After stratifying by gender, the direction of associations did not change except for eczema in females, and for eczema in males (Table S2). After stratifying for the father’s education, direction for rhinitis in the lower education group was reversed, and no statistical significance was observed (Table S3). To better compare the NDVI measure and proximity to parks, a similar analysis was done for the NDVI measure, i.e., the ORs were calculated for participants within different quartiles of NDVI values, referenced to the lowest NDVI quartile. No statistically significant results were observed, as shown in Table S4.

The PM2.5 concentrations, pets in the home, dampness and mold in the child’s room were tested as additional potential confounders (Table S5). The results for NDVI or distance to parks did not change after additional adjustment for each of these variables. 

## 4. Discussion

In the first study in Asia to study the effects of greenspace on respiratory and allergic diseases, we did not observe an association between NDVI and asthma or allergic outcomes. However, in both crude and fully-adjusted models, we observed that living far from a park was associated with decreased odds of current and ever asthma. Pneumonia, rhinitis, and eczema associations were directionally similar but did not reach significance at the *p* < 0.05 level. These associations were unchanged after additional adjustment for air pollution, pets in the home, and dampness and mold issues. 

While most studies in developed countries have observed increasing levels of average NDVI within larger buffer areas, the median values of our data stayed approximately the same within different areas, which may reflect different urban forms in Chinese cities compared to cities in Western countries [7]. Suzhou consists of an Ancient City (urban core), two new satellite districts (industrial parks), and two suburban districts. In the Ancient City, there are over two hundred traditional houses dating back to the Ming and Qing dynasties. This area continues to be used for residential and retail purposes. The newer industrial park communities were built to permit modernization, expand the traditional city, and reduce the urban density in the Ancient City. The rapid improvement of the existing city infrastructure, with a more modern infrastructure and increased land for newer industries is the centerpiece of Suzhou’s strong economic base. The two suburban areas are a mix of residential use, ecological agriculture, and traditional industry. Overall, modernization of this historic city offers a variety of housing types and neighborhood infrastructures for studying the relationships among health and aspects of the built environment. 

We did not observe associations between NDVI on any of our outcomes (Table 3 and Table S4). This is consistent with findings from other studies conducted in Western countries. Pilat et al. [6] observed no association between NDVI and asthma in Texas, USA, which they attributed to a small sample size and the spatial scale of the exposure measures used. Dadvand et al. [7] also observed no association between asthma or rhinitis and NDVI in a Barcelona-based cohort, even with a more spatially resolved exposure measure. A study in Portugal done by Ayres-Sampaio, however, showed that low NDVI was associated with increased asthma hospitalizations [28]. They attributed this to the fact that low NDVI areas were concentrated in highly urbanized areas and were usually correlated with high levels of air pollution. We tested the correlation between air pollution level and NDVI in our dataset and found the correlations were weak (Table S6). We also assessed if our finding were confounded by PM2.5 concentrations, (Table S5), and there was no evidence of confounding. This may reflect the difference in urban forms in China compared to urban forms in Portugal, i.e., air pollution is not necessarily linked with less vegetation. Further investigation of these potential confounders is needed in a broad range of urban and rural settings. A Canadian study by Sbihi et al. [29] reported that traffic pollution increased the chance of chronic asthma while there was no association with greenspace. 

As NDVI is a measure of various types of vegetation, using more specific measures such as parks may be helpful to identify the mechanism of how this type of green space affects respiratory health. We found living far from a park to be a protective factor for asthma. This result was similar to a Spanish study that found living close to a park was associated with increased doctor-diagnosed asthma [7]. The study by Fuertes et al. [8] comparing seven birth cohorts also revealed mixed trends for the association between residential greenness and rhinitis, suggesting the associations might be confounded by location-specific factors. 

The exact mechanisms behind the associations between greenspace and asthma and allergic outcomes remains to be elucidated [8]. One explanation is that living far from a park reduces asthma rates due to decreased exposure to pollen in these spaces. Exposure to tree pollen was found to be associated with an increased IgE response, which could lead to allergic symptoms [5]. Codispoti et al. [30] reported being born in seasons with high releases of tree or grass pollen increased the risk of allergic symptoms by a factor of three, which may also suggest an adverse effect due to pollen exposure. On the other hand, greenspace is believed to affect atopic outcomes through exposure to diverse microbes, with the mechanism explained by the hygiene hypothesis and the biodiversity hypothesis [10,11,12,31,32,33]. The hygiene hypothesis emphasizes the effect of early-life exposure factors on the development of allergic outcomes. Factors that increase the diversity of microbiome exposure are believed to increase immune tolerance and reduce the risk of developing atopic outcomes. The biodiversity hypothesis extends the hygiene hypothesis by pointing out that the loss of biodiversity also leads to decreased diversity of microbes, potentially causing allergic symptoms to increase. Based on these hypotheses, greenspace may be protective of atopic diseases, assuming that sufficient biodiversity is maintained. In our study we do not have sufficient information on at what age the adolescent started to use parks or their frequency of usage, or the biodiversity of the parks, so we cannot speculate whether the hygiene hypothesis or biodiversity hypothesis are relevant. Our results suggest living far from parks might decrease the risk of allergic symptoms. Given that parks are important urban amenities, the underlying explanation for our observed associations should be further studied to determine ways to ameliorate any deleterious aspects. 

Eczema and pneumonia were seldom addressed in previous studies of the effects of greenspace. We did not observe statistically significant associations between our exposure and eczema or pneumonia. The development of eczema is believed to be related to early life exposure to different pathogens [34]. Yet, not all microbial exposure protects against eczema, and it is still unclear which types of infections are protective or risk factors for eczema [35,36]. In a recent World Health Organization (WHO) bulletin, the risk factors for pneumonia were summarized and most of the *definite* risk factors were related to factors other than the environment (e.g., malnutrition, lack of immunization, non-exclusive breastfeeding) [37]. While indoor and outdoor air pollution has also been identified as a risk factor, few studies have likely examined the role of other environmental factors such as greenspace. Our findings suggest that greenspace—as an environmental factor—does not have an effect on childhood pneumonia.

This study has a number of limitations. Like other cross-sectional studies, we are unable to determine a causal relationship between our exposures and health outcomes. We also cannot rule out the possibility of self-selection bias. It is possible that parents of adolescents diagnosed with respiratory or allergic diseases may move to an area closer to parks due to their perceived health benefits. We do not have enough information on the moving behavior of the families to confirm that the exposure was the same as the study period for all the families at the time when the diseases (rhinitis, pneumonia, eczema) happened. In future studies, it is important to obtain moving information to assess exposure–outcome relationships, especially to be able to examine the impacts of early life exposures. Another limitation is that we were only able to examine the impacts of NDVI and distance to parks in the year of the questionnaire, which by definition was after the time of diagnosis for each child. This may not be the etiologically relevant time period of exposure for these outcomes, which may partially explain our findings. Studies with exposures over longer time periods would be needed to clarify these issues. We used home addresses for calculating exposure, which does not take into account exposures in a school setting. A commonly cited issue with the use of NDVI and distance to parks is that neither measure is able to take into account the quality or accessibility of the greenspace [7,14]. Euclidean distance is used, which might not accurately reflect the distance to access the parks. Also, we did not take the size of parks into consideration, which may potentially lead to inaccuracy of exposure assessment. Lastly, the urban areas in our study and in previous studies are heterogeneous in terms of vegetation types, urban form, and geography, and there is little information available on what specific type of tree or pollen might be the primary cause. Our results may be shaped by unmeasured co-incidental urban environmental exposures or characteristics of the study population that determine the risk of allergic or respiratory outcomes. As we do not have perfect knowledge about geological context around each home addresses, the analyses might be affected by the uncertain geographic context problem [38]. Another limitation was the limited area-level SES information available to us including direct data on family income; we used parental education as out measure of SES, which might lead to residual confounding. The different urban forms in Suzhou suggests that this study might be generalizable to other cities in China, but more studies are needed, especially in inland areas where urban forms are more different than coastal areas. Our school-based sampling, although more efficient, may increase risk of bias, if the schools that participated in the study are not representative of the city, resulting in an increased sampling error.

Our study has several strengths. This was the first study in China to explore the impacts of NDVI and distance to parks on respiratory and allergic outcomes in adolescents. Our large sample size, geographically diverse sample, and validated outcomes made for a robust study of these associations, while controlling for a number of potentially important confounders. Lastly, we were able to examine the impacts of multiple measures of exposure.

## 5. Conclusions

We conducted the first study in China that explores the relationship between various allergic outcomes and exposure to neighborhood greenness and green spaces. We found that NDVI values did not vary with buffer size, which might be attributable to the different urban forms in Chinese cities when compared to cities in Western countries. We did not observe associations between NVDI and respiratory and allergic outcomes. Living closer to parks appeared to be a risk factor for asthma. Our results suggest that although greenspace is an important element of urban infrastructure, the associations between greenspace and health remain poorly understood and we need to understand the specific mechanisms that govern these linkages. 

## Figures and Tables

**Table 1:** Distribution of demographic characteristics and prevalence of outcomes.

		Overall % (*N* = 5643)	% of Missing Data
Adolescents’ gender	Male	51.6	2.4
Adolescents’ age	12 and below	10.2	11.0
	13	43.8	
	14	40.3	
	15 and above	5.7	
Parental education	Primary school	5.8	5.1
	Middle school	25.7	
	High school	24.5	
	College and above	44.0	
Family asthma history	Yes	12.9	5.9
ETS ^a^ at home	Yes	49.4	2.7
Current asthma	Yes	3.9	10.5
Ever asthma	Yes	9.8	4.7
Ever pneumonia	Yes	20.7	4.5
Ever rhinitis	Yes	20.2	5.2
Ever eczema	Yes	18.5	6.0

^a^ Environmental tobacco smoke.

**Table 2:** Overall and season-specific normalized difference vegetation index (NDVI) values (median values and interquartile range (IQR)).

	Buffer 100 m	Buffer 200 m	Buffer 500 m	Buffer 1000 m
Overall median (IQR) NDVI	0.187 (0.101)	0.187 (0.088)	0.190 (0.073)	0.187 (0.055)
Spring median (IQR) NDVI	0.174 (0.095)	0.176 (0.081)	0.180 (0.059)	0.173 (0.051)
Summer median (IQR) NDVI	0.298 (0.095)	0.294 (0.091)	0.297 (0.078)	0.297 (0.061)
Fall median (IQR) NDVI	0.139 (0.113)	0.139 (0.095)	0.143 (0.071)	0.140 (0.056)
Winter median (IQR) NDVI	0.138 (0.090)	0.139 (0.082)	0.140 (0.066)	0.140 (0.045)

**Table 3:** Odds ratios of association between respiratory and allergic outcomes and NDVI IQR in different buffer areas. ^a^

		100-m Buffer	200-m Buffer	500-m Buffer	1000-m Buffer
Current Asthma	Crude	1.06 (0.91, 1.23)	1.01 (0.83, 1.24)	0.95 (0.75, 1.19)	0.97 (0.79, 1.20)
	Fully-adjusted ^b^	1.10 (0.93, 1.29)	1.09 (0.86, 1.38)	1.01 (0.77, 1.31)	1.02 (0.80, 1.31)
Ever Asthma	Crude	1.02 (0.94, 1.12)	1.00 (0.89, 1.14)	0.96 (0.83, 1.11)	0.95 (0.84, 1.08)
	Fully-adjusted ^b^	1.03 (0.93, 1.13)	1.01 (0.88, 1.16)	0.95 (0.81, 1.12)	0.94 (0.82, 1.08)
Ever Pneumonia	Crude	0.97 (0.91, 1.04)	0.96 (0.88, 1.06)	0.94 (0.84, 1.05)	0.95 (0.86, 1.05)
	Fully-adjusted ^b^	0.97 (0.91, 1.04)	0.95 (0.87, 1.05)	0.92 (0.82, 1.04)	0.94 (0.85, 1.05)
Ever Rhinitis	Crude	0.99 (0.92, 1.06)	0.99 (0.90, 1.09)	0.97 (0.86, 1.09)	0.99 (0.89, 1.10)
	Fully-adjusted ^b^	0.96 (0.90, 1.04)	0.95 (0.86, 1.06)	0.93 (0.82, 1.06)	0.96 (0.86, 1.07)
Ever Eczema	Crude	1.03 (0.96, 1.11)	1.04 (0.94, 1.16)	1.05 (0.92, 1.19)	1.04 (0.93, 1.16)
	Fully-adjusted ^b^	1.01 (0.94, 1.09)	1.01 (0.91, 1.13)	1.04 (0.91, 1.19)	1.03 (0.92, 1.16)

^a^ The IQR for the 100-m buffer, 200-m buffer, 500-m buffer, and 1000-m buffer were 0.101, 0.088, 0.073, and 0.055, respectively. ^b^ Fully-adjusted model adjusted for child’s age and sex, ETS at home, parental education, and parental history of asthma.

**Table 4:** Crude and fully-adjusted odds ratios (ORs) (95% CIs) of targeted outcomes associated with quartiles of distance from a park.

		1st Quartile (<600 m)	2nd Quartile	3rd Quartile	4th Quartile
			(600–903 m)	(903 m–1348 m)	(>1348 m)
Current Asthma	Crude	Reference	1.00 (0.66, 1.51)	1.03 (0.68, 1.57)	0.60 (0.37, 0.98) *
	Fully-adjusted ^a^	Reference	0.96 (0.62, 1.49)	0.96 (0.61, 1.50)	0.58 (0.35, 0.99) *
Ever Asthma	Crude	Reference	1.03 (0.79, 1.34)	0.99 (0.75, 1.30)	0.68 (0.50, 0.93) *
	Fully-adjusted	Reference	1.02 (0.77, 1.35)	0.93 (0.70, 1.24)	0.70 (0.50, 0.96) *
Ever Pneumonia	Crude	Reference	0.99 (0.81, 1.21)	0.90 (0.73, 1.11)	0.87 (0.70, 1.07)
	Fully-adjusted	Reference	1.00 (0.81, 1.24)	0.90 (0.73, 1.12)	0.92 (0.74, 1.15)
Ever Rhinitis	Crude	Reference	1.24 (1.01, 1.52)	1.14 (0.92, 1.40)	0.94 (0.75, 1.18)
	Fully-adjusted	Reference	1.16 (0.93, 1.45)	1.12 (0.89, 1.40)	0.97 (0.76, 1.24)
Eczema	Crude	Reference	1.04 (0.85, 1.28)	0.96 (0.77, 1.19)	0.85 (0.68, 1.07)
	Fully-adjusted	Reference	0.99 (0.79, 1.23)	0.92 (0.74, 1.16)	0.86 (0.68, 1.10)

^a^ Adjusted for child’s age and sex, ETS at home, parental education, and parental history of asthma. * statistically significant.

## Supplementary Materials

The following are available online at https://www.mdpi.com/1660-4601/16/6/991/s1. Table S1. List of parks, their addresses, latitude and longitude. Table S2. Correlations between air pollutant concentrations (including PM10, PM2.5, NO_2_, SO_2_, O_3_ and CO) and average NDVI within different buffers (100 m, 200 m, 500 m, 1000 m). Table S3. Testing confounding effect of PM2.5, pet ownership, dampness and mold by including them in the full model and compare the odds ratios with the original full model. Table S4. Fully-adjusted^a^ ORs (95% CIs) of targeted outcomes associated with quartiles of NDVI values. Table S5. Fully-adjusted^a^ ORs (95% CIs) of targeted outcomes associated with quartiles of distance from a park, stratified by gender. Table S6. Fully-adjusted^a^ ORs (95% CIs) of targeted outcomes associated with quartiles of distance from a park, stratified by father education.
