# Supplementary material for: Effect of Residential Greenness and Nearby Parks on Respiratory and Allergic Diseases among Middle School Adolescents in a Chinese City
Source: Int J Environ Res Public Health. 2019 Mar 19;16(6):991. doi: 10.3390/ijerph16060991 (PMC6466062; doi:10.3390/ijerph16060991)
Supplement: Supplementary file 1 [file ijerph-16-00991-s001.pdf]

Table S1. List of parks, their addresses, latitude and longitude

ID	店名	地址	Addresses	latitude	longitude
1	苏州乐园	江苏省苏州市虎丘区长江路 397 号	江苏省苏州市虎丘区长江路 393 号	31.29601	120.5448
2	荷塘月色湿地公园	苏州市相城区太阳路西 4575 号(近黄桥下庄)	江苏省苏州市相城区 X303(太阳路)	31.4109	120.5792
3	木渎公园	江苏省苏州市吴中区中山西路	江苏省苏州市吴中区 X206(中山西路)	31.25855	120.5117
4	昆山市城市生态森林公园	马鞍山西路 888 号	江苏省苏州市昆山市马鞍山西路 888 号	31.39225	120.9053
5	苏州公园	沧浪区公园路 111 号	江苏省苏州市姑苏区五卅路 130	31.30675	120.6234
6	虎丘湿地公园	苏虞张公路和阳澄湖西路交界处附近	江苏省苏州市相城区阳澄湖西路	31.36717	120.5779
7	新区公园	塔园路 279 号	江苏省苏州市虎丘区 Y205(珠江路)	31.30448	120.533
8	苏州太湖国家湿地公园	高新区镇湖市湖绣品街 1 号	江苏省苏州市虎丘区前景北路	31.32211	120.3565
9	虞山公园	苏州市常熟市	江苏省苏州市常熟市虞山中路	31.66276	120.7137
10	苏州动物园	江苏省苏州平江区白塔东路 5-1 号	江苏省苏州市姑苏区北仓街	31.32311	120.6343
11	潜龙渠公园	吴江市盛泽新城	江苏省苏州市吴江区澜溪二路	30.91408	120.6066
12	中央公园	星明街与苏州大道西交汇处	江苏省苏州市吴中区星兰街	31.31625	120.6581
13	吴江公园	吴江松陵镇仲英大道(近笠泽路)	江苏省苏州市吴江区笠泽路	31.15764	120.6308
14	沙湖生态公园	苏州市工业园区内	江苏省苏州市吴中区星华街	31.33344	120.7637
15	苏州太湖湖滨国家湿地公园	胥香路与环太湖大道交汇处	-	31.32211	120.3565
16	暨阳湖生态园	暨阳湖生态园区内	江苏省苏州市张家港市暨阳湖大道	31.84699	120.5321
17	金仓湖公园	江苏省苏州市太仓市太沙路与浏双线交叉口北侧 50 米	江苏省苏州市太仓市 X303(太沙线)	31.51073	121.0933
18	桐泾公园	江苏省苏州沧浪区桐泾南路 1 号	江苏省苏州市姑苏区解放东路	31.28452	120.5979
19	阳澄湖水上公园	苏州沧浪区巴城镇巴解路阳澄湖水上公园	江苏省苏州市昆山市巴解路	31.45642	120.8515
20	亭林园	江苏省苏州市昆山市马鞍山东路 1 号	江苏省苏州市昆山市 Y001(马鞍山东路)	31.39273	120.9451
21	何山公园	江苏省苏州吴江市新区长江路 461 号	江苏省苏州市虎丘区何山路	31.30491	120.5382
22	大白荡城市生态公园	苏州市浒墅关经济开发区鸿福路 20	江苏省苏州市虎丘区鸿禧路	31.35171	120.5099
23	桂花公园	竹辉路 1 号(竹辉桥东堍)	江苏省苏州市姑苏区竹辉桥	31.29484	120.6396
24	白塘生态植物园	苏州市工业园区	江苏省苏州市吴中区百合北路	31.3329	120.7341
25	镜湖公园	吴江盛泽镇如意路(近 227 省道)	江苏省苏州市吴江区红安路	30.9193	120.67
26	大阳山国家森林公园	苏州市高新区浒关经济开发区山神湾路	江苏省苏州市虎丘区山神湾路	31.3501	120.4617
27	耦园	苏州市姑苏区仓街小新桥巷 6 号(近平江路)	江苏省苏州市姑苏区仓街	31.31828	120.6341
28	芦荡湖湿地公园	吴江芦荡路以北,中心路以南,鲈乡南路以东,中山南路以西	江苏省苏州市吴江区芦荡路	31.11785	120.6336
29	柏庐公园	站北二路与柏庐南路交叉口附近	江苏省苏州市昆山市柏庐南路 900 号	31.35577	120.9556
30	阳澄湖湖滨生态体育公园	工业园区亭盛街 50 号西侧(近唯盛邻里商务酒店)	江苏省苏州市吴中区跨澄路	31.3747	120.7035
31	鳄鱼谷	昆山长江南路(恒丰路口)	江苏省苏州市昆山市 S224(长江南路)	31.26792	120.9551
32	广济公园	山塘风景区旁	江苏省苏州市姑苏区广济路 133 号	31.3185	120.5942
33	昆山体育生态公园	周市镇永共路与迎宾东路交叉口附近	江苏省苏州市昆山市 X302(迎宾东路)	31.4547	120.9834
34	千灯公园	新虹路与汉昆路交叉口西侧	江苏省苏州市昆山市汉昆路	31.25099	121.0174
35	张家港公园	其他地区港城大道沙洲西路口(近港城大道)	江苏省苏州市张家港市 X204(港城大道)	31.86463	120.525
36	黄埭镇三岛公园	苏州市相城区	江苏省苏州市相城区 X302(太东公路)	31.44813	120.5385
37	郑和公园	南环路与滨江大道交叉口东侧 200 米	江苏省苏州市太仓市南环路	31.60359	121.2407
38	漕湖湿地公园	放桥路与环湖路交界处附近	江苏省苏州市相城区环湖路	31.4866	120.5935
39	运河公园	三香路 1346 号	江苏省苏州市姑苏区运河路	31.30209	120.5689

40	花溪公园(花园路)	花桥镇花桥镇花园路	江苏省苏州市昆山市 Y453(花园路)	31.29778	121.0883
41	梁丰生态园	其他地区东二环路西侧(沙洲东路南侧)	江苏省苏州市张家港市南苑东路 2	31.85561	120.5691
42	江苏虞山国家森林公园(寺路街)	近郊常熟市虞山北路中路南路	江苏省苏州市常熟市虞山中路	31.67548	120.7007
43	白鹭园	工业园区翠薇街 101 号(近独墅湖高校区、独墅湖隧道)	江苏省苏州市吴中区翠薇街	31.27344	120.7158
44	苏州花卉植物园	生态园园中大道	江苏省苏州市相城区园中大道	31.42668	120.5958
45	荷花公园	工业园区斜塘联丰广场	江苏省苏州市吴中区松涛街	31.29957	120.7289
46	白洋湾公园	鹿山路与城北西路交叉口	江苏省苏州市姑苏区鹿山路	31.342	120.5623
47	人民公园	城厢镇县府西街 40 号(公园弄口)	江苏省苏州市太仓市县府西街 50 号	31.44842	121.0965
48	东湖公园	吴中区城南街道东湖社区居民委员会附近	江苏省苏州市吴中区盛城路	31.22736	120.6237
49	魔法奇园	桐泾北路 218 号（来客茂时尚生活中心 2 楼）	江苏省苏州市姑苏区枫桥路 361	31.31471	120.5851
50	沙洲公园	杨舍镇公园路(暨阳路口)	江苏省苏州市张家港市暨阳中路	31.87145	120.547
51	莲池湖公园	工业园区阳澄湖半岛莲池湖路(近重元寺)	江苏省苏州市吴中区莲池湖路	31.4136	120.7811
52	珍珠湖公园	渭塘镇玉盘路 129 号小四川旁	江苏省苏州市相城区玉盘路 113	31.46844	120.6563
53	玉山公园	南投县水里乡中山路一段 300 号	江苏省苏州市虎丘区玉山路	31.28756	120.5433
54	方洲公园	工业园区方洲路(近方洲邻里中心)	江苏省苏州市吴中区方洲路	31.31773	120.7418
55	独墅湖生态公园	工业园区郭新东路与东方大道交叉口附近	江苏省苏州市吴中区赏湖路	31.2506	120.7042
56	芦墟大渠荡生态公园	吴江芦墟镇芦莘大道	江苏省苏州市吴江区临沪东路	31.02948	120.8469
57	盛泽湖月季公园	相城盛泽湖北、环湖西路东	江苏省苏州市相城区盛泽荡路	31.45864	120.704
58	松陵公园	流虹路 271 号	江苏省苏州市吴江区流虹路 267	31.16035	120.6423
59	丹桂园主题公园	张浦镇阳光中路 1 号	江苏省苏州市昆山市 S343(阳光西路)	31.28001	120.9106
60	盛泽广场	吴江盛泽镇姚家坝桥西侧	江苏省苏州市吴江区市场东路 182 号	30.90578	120.6488
61	时代公园	昆山其他开发区时代公园	江苏省苏州市昆山市太湖中路	31.38295	121.0326
62	目澜洲公园	舜新南路 1933	江苏省苏州市吴江区舜新南路	30.89653	120.654
63	湘园	城中路与沈周路交汇处	江苏省苏州市相城区沈周路 1 号-3 幢	31.49288	120.7287
64	元和公园	蠡中路与芙蓉街交界处	江苏省苏州市相城区采莲路	31.39888	120.6187
65	阳澄湖公园	西北部的阳澄湖畔	江苏省苏州市昆山市环城西路	31.38901	120.8374
66	唯亭公园	戈港路与金陵西路交汇处西	江苏省苏州市吴中区金陵西路	31.36064	120.7806
67	石浦公园	苏州市昆山市	江苏省苏州市昆山市振石路	31.25799	121.0434
68	灵峰公园	樊店寺院旁	江苏省苏州市相城区聚乐路	31.52994	120.6136
69	月季园	相城区盛泽湖休闲度假区(近渭中路)	江苏省苏州市相城区盛泽荡路	31.45887	120.7046
70	小义园	苏州市常熟市	江苏省苏州市常熟市红旗路	31.70481	120.6878
71	上方山国家森林公园	吴中区横塘镇横越路 47 号上方山国家森林公园	江苏省苏州市虎丘区横越路 47 号	31.25393	120.582
72	张陵公园	江苏省苏州市吴中区甪直镇西南 1.5 公里处	江苏省苏州市吴中区鸿福路	31.25605	120.8591
73	横山公园	滨河路 87 号	江苏省苏州市虎丘区金屋路	31.27465	120.5667
74	聚沙园	梅李镇梅李镇梅东路 1 号	江苏省苏州市常熟市聚沙路	31.70754	120.872
75	滨河公园	柳州路与滨河路交叉口	江苏省苏州市太仓市滨河路 232 号	31.44198	121.1263
76	中心广场	城厢镇浮桥镇中路	江苏省苏州市姑苏区人民路 3188 号	31.34496	120.61
77	黎里公园	吴江市黎里镇人民中路 80 号	江苏省苏州市吴江区人民中路	30.99637	120.7093
78	曾赵园	其他地区西南隅翁府前(近第一人民医院)	江苏省苏州市常熟市环城西路	31.64369	120.7312
79	彩香公园	干将西路 1099 号(干将西路与桐泾南路交汇处)	江苏省苏州市姑苏区彩香路	31.30208	120.5858
80	诺贝尔湖公园	高新区科技城科研路	江苏省苏州市虎丘区科研路	31.33986	120.4289
81	黄桥公园	永方路与春申湖西路交界处	江苏省苏州市相城区永方路	31.38073	120.5865

82	大阳山植物园景区	江苏省苏州市新区浒墅关经济开发区阳山环路 8 号	江苏省苏州市虎丘区 Y266(南阳山路)	31.32915	120.4515
83	江滨公园	星湖街与车郭路交汇处	江苏省苏州市吴中区星湖街	31.24144	120.7305
84	三香公园	三香路 108-1 号	江苏省苏州市姑苏区三香路 77 号	31.30195	120.602
85	城市公园	长江中路与震川东路交叉口东	江苏省苏州市昆山市 S224(长江中路)	31.3821	120.9759
86	巴城湖公园	巴城镇	江苏省苏州市昆山市 Y002(临湖路)	31.46279	120.8586
87	运动公园	东林陶瓷有限公司东北侧	江苏省苏州市吴中区环湖路	31.23898	120.6749
88	百花洲公园	百花洲西大街 105 号（近学士街）	江苏省苏州市姑苏区吉庆街	31.29721	120.6085
89	锦溪苑	工业园区榭雨街 66 号南侧	江苏省苏州市吴中区锦溪街	31.30715	120.759
90	范蠡公园	苏蠡路与澄湖路交汇处	江苏省苏州市吴中区澄湖路	31.24308	120.6124
91	迎湖公园	312 国道	江苏省苏州市相城区腾飞东路 8 号	31.42382	120.4273
92	浏河公园	太仓市浏河镇郑和南路 36 号	江苏省苏州市太仓市郑和南路 65 号	31.50358	121.2637
93	景秀园	山泉湖生态园斜对面	江苏省苏州市常熟市环湖北路	31.65528	120.6908
94	蒹葭生态园	陆家镇 404 乡道	江苏省苏州市昆山市 Y404	31.30892	121.0352
95	湖滨大道	工业园区星港街(星州街)	江苏省苏州市吴中区星州街	31.31574	120.6778
96	陆慕公园	阳澄湖西路	江苏省苏州市相城区阳澄湖西路 32 号	31.3645	120.6121
97	张林园	甪直风景区甪直镇张林村甫澄中路(近东庄路)	江苏省苏州市吴中区 X201(甫澄中路)	31.25222	120.8625
98	汾湖公园	吴江区芦墟镇汾湖园路 301 号	江苏省苏州市吴江区汾湖园路	31.00999	120.8291
99	滨江公园	江苏省苏州市常熟市	江苏省苏州市常熟市新溪路	31.72304	120.9233
100	星海公园	园区星海街 200 号(苏绣路口)	江苏省苏州市吴中区星海街	31.31224	120.6688
101	苏州格林乡村公园	七都镇庙港莘七线格林乡村公园(近太浦闸)	江苏省苏州市吴江区莘七线	31.01579	120.4858
102	中心湖公园	江苏省苏州市昆山市 y869	江苏省苏州市昆山市 Y004(萧林西路)	31.41018	120.8916
103	白塔公园	皮市街与白塔西路交叉处	江苏省苏州市姑苏区白塔西路 47 号	31.3197	120.6205
104	索山公园	苏州高新区狮山路	江苏省苏州市虎丘区狮山路 321 号	31.2943	120.5474
105	东方蛇园	辛庄镇辛庄镇界善村	江苏省苏州市常熟市隆力奇大道	31.50436	120.658
106	水秀公园	玉山镇水秀路	江苏省苏州市昆山市水秀路	31.40311	120.9398
107	紫薇园	相城区凤阳路	江苏省苏州市相城区湘洲路	31.48624	120.7054
108	观前公园	富仁坊巷新埃及城时尚百货附近	江苏省苏州市姑苏区北局一弄 9 号	31.31118	120.6208
109	阳澄湖公园	相城区阳澄湖汽车站对面(阳澄湖镇田泾管理区管委会南)	江苏省苏州市昆山市环城西路	31.38901	120.8374
110	七都太湖湿地公园	苏州市吴中区	江苏省苏州市吴中区莘七线	30.9662	120.4063
111	城西公园	其他地区云盘路	江苏省苏州市张家港市云盘路 34 号	31.87032	120.5315
112	虞山公园动物园	虞山森林公园	江苏省苏州市常熟市虞山中路	31.65125	120.7324
113	垂虹遗址公园	吴江松陵镇东门外的垂虹桥	江苏省苏州市吴江区花园路 2466 号	31.15889	120.6448
114	晨曦公园	昆山其他太湖中路	江苏省苏州市昆山市震川东路	31.38276	121.0381
115	翠坊公园	香溪路	江苏省苏州市吴中区乐园路	31.25512	120.516
116	太湖新天地生态休闲公园	中信太湖城知湖轩旁	江苏省苏州市吴中区 X204(环太湖大道)	31.21749	120.4209
117	孙武公园	高新区邓尉路 23 号	江苏省苏州市虎丘区邓尉路 23 号	31.30037	120.5376
118	景枫公园	昆山其他玉山镇开发区庆丰东路	江苏省苏州市昆山市庆丰东路 150 号	31.35568	120.9928
119	通江公园	苏州市张家港市	江苏省苏州市张家港市迎宾路	31.8776	120.807
120	沙葛公园	苏州市昆山市陆家镇金阳西路	江苏省苏州市昆山市泾新路	31.34541	120.9889
121	黄浦公园	江苏省昆山市前进东路与黄浦江路交叉口	江苏省苏州市昆山市 X203(黄浦江中路)	31.38403	121.0056
122	越来溪公园	苏州市吴中区文溪路	江苏省苏州市吴中区文溪路	31.20527	120.5874
123	五龙桥公园	苏州市吴中区	江苏省苏州市吴中区太湖东路	31.26337	120.6279

124	太湖西山国家地质公园	苏州市吴中区金庭镇缥缈路太湖西山国家森林公园缥缈峰景区内	江苏省苏州市吴中区飘渺路	31.11819	120.2468
125	小康公园	小康大桥旁	江苏省苏州市常熟市 Y624	31.61494	120.8201
126	西塘公园	城北西路与虎阜路交界处西侧	江苏省苏州市姑苏区西塘河路	31.34252	120.5794
127	大庄公园	003 村道	江苏省苏州市相城区大庄桥	31.39142	120.5516
128	震泽公园	吴江震泽镇中心大桥堍(近老震泽汽车站)	江苏省苏州市吴江区公园路	30.91629	120.4958
129	金浦公园	苏州市工业园区胜浦街道	江苏省苏州市吴中区振胜路	31.30874	120.8187
130	城北湿地公园	苏州市太仓市	江苏省苏州市太仓市太平北路	31.46702	121.0992
131	阳澄湖公园(环城西路)	巴城镇西北部的阳澄湖畔(近费尔蒙酒店)	江苏省苏州市昆山市马鞍山西路	31.38888	120.8369
132	憩园	玉山镇花园路 856 号	江苏省苏州市昆山市花园路 846 号	31.39985	120.9469
133	太丰西庐生态湿地公园	昆太路太丰西庐生态湿地公园附近	江苏省苏州市太仓市庙浜路	31.43102	121.0656
134	澹台湖公园(迎春南路)	太湖东路 363 号	江苏省苏州市吴中区迎春南路	31.26106	120.6372
135	朴园	校场桥路 8 号	江苏省苏州市姑苏区校场桥路 8 号	31.3261	120.611
136	半园	白塔东路 60 号	江苏省苏州市姑苏区白塔东路 56 号	31.32173	120.6294
137	碧溪公园	扬子江大道与西聚福路交汇处附近	江苏省苏州市常熟市碧溪西路	31.73589	120.9316
138	陈家浜公园	苏州市昆山市	江苏省苏州市昆山市大通路	31.35901	121.0827
139	虞园	虞山镇青墩塘路	江苏省苏州市常熟市海虞北路 24 号	31.66078	120.7509
140	昆山市花卉博览园	昆山其他阳光东路	江苏省苏州市昆山市金丰路	31.27516	120.9694
141	湖滨公园	芙蓉街 9 号	江苏省苏州市吴中区星州街	31.31648	120.6783
142	江苏震泽省级湿地公园	吴江震庙公路	江苏省苏州市吴江区 C807	30.94025	120.498
143	合兴公园	昆山其他合兴西路	江苏省苏州市昆山市团圆弄	31.3736	120.9487
144	莲湖公园	巴城镇古城路	江苏省苏州市昆山市 X205(古城北路)	31.38481	120.8756
145	香樟园	苏州市工业园区	江苏省苏州市吴中区梧桐街	31.21837	120.61
146	体育公园	工业园区东南大学软件学院旁	江苏省苏州市吴中区崇文路	31.27191	120.7372
147	清水岸生态公园	美人腿（近阳澄湖）	江苏省苏州市相城区 X201(澄林路)	31.42623	120.7447
148	中央公园	苏州市昆山市	江苏省苏州市吴中区星兰街	31.31625	120.6581
149	中塘公园	工业园区金鸡湖东岸星湖街与中新大道东交界处(近湖东邻里中心)	江苏省苏州市吴中区中新大道东	31.31033	120.7225
150	桃园	学士街 243 号附近	江苏省苏州市姑苏区学士街 224 号	31.30516	120.608
151	滨江体育公园	其他地区沿江开发区	江苏省苏州市常熟市江南大道	31.74208	120.9227
152	黄金港公园	葑亭街青苑新村四区附近	江苏省苏州市吴中区青灯街	31.36717	120.7794
153	琅环公园	玉山镇西街	江苏省苏州市昆山市江苏省西街 27 号	31.38232	120.9503
154	淀山湖体育公园	苏州市昆山市	江苏省苏州市昆山市 Y612(中市路)	31.17834	121.0202
155	风之园	苏州市吴中区苏州工业园区金鸡湖景区内	江苏省苏州市吴中区中新大道西	31.30808	120.6798
156	上海野生动物园（昆山上车点）	玉山镇人民北路 99 号	江苏省苏州市昆山市人民北路 99 号	31.38361	120.9535
157	尹山国际汽车城汽车主题公园	尹南路	江苏省苏州市吴中区 S302(吴东路)	31.23558	120.6601
158	街心公园	快鸭港路附近	江苏省苏州市吴江区镇南路 1478	30.91189	120.5021
159	三里桥生态公园	吴江北门街路附近香江花园东门口	江苏省苏州市吴江区交通南路 16 号	31.16845	120.6489
160	板桥财富广场	其他地区新区板桥南京东路	江苏省苏州市太仓市四通路	31.49366	121.1263
161	新吴园	张浦镇新吴街与宝觉街交叉口西侧	江苏省苏州市昆山市宝觉街 36 号	31.28739	120.9434
162	水之韵城市休闲文化公园	昆山其他前进路至震川路太湖路至夏驾河路	江苏省苏州市昆山市夏驾南路 14 号	31.38487	121.0421
163	老太庙	苏州市吴江市	江苏省苏州市吴江区庙震路	30.99156	120.471
164	青年公园	高新区阳山公寓旁	江苏省苏州市虎丘区阳山东路	31.34715	120.4735

165	湖心熙园	苏州市吴中区	江苏省苏州市吴中区阳澄环路	31.4123	120.7803
166	唯亭生态体育公园	苏州市吴中区	江苏省苏州市吴中区运澄路	31.38282	120.7166
167	御亭公园	御亭路与鹤溪路交界处附近	江苏省苏州市相城区御亭路	31.4226	120.445
168	海德公园北门自行车租赁点	苏州市吴中区	江苏省苏州市吴中区林泉街 566 号	31.27614	120.7418
169	金华园	昆山其他江浦南路与 C307 交叉口西侧	江苏省苏州市昆山市 C307	31.30926	120.9151
170	澄园	澄阳路与蠡塘河路交界处附近	江苏省苏州市相城区蠡塘河路	31.39074	120.6415
171	学府公园	华元路与济学路交界处附近	江苏省苏州市相城区济学路	31.37914	120.6655
172	东吴国家森林公园	苏福公路 44 号林场内	江苏省苏州市吴中区 Y413(兵圣路)	31.26693	120.4303
173	长江公园	昆太路与长江北路交叉口北	江苏省苏州市昆山市 S224(长江北路)	31.39504	120.9731
174	夕露园	巴城镇 156 村道	江苏省苏州市昆山市民权路	31.39229	120.8313
175	鹤园	镇北路与公园街交叉口南侧	江苏省苏州市姑苏区平江路 298-2 号	31.32261	120.6281
176	友联公园	长吴路 125 号	江苏省苏州市姑苏区长吴路 121 号	31.27458	120.6052
177	车塘农民乐园	苏州市昆山市	江苏省苏州市昆山市 X203	31.33025	121.0215
178	灵岩牡丹园	吴中区灵岩山南麓	江苏省苏州市吴中区穹灵路	31.26638	120.495
179	西桥公园	玉山镇震川西路	江苏省苏州市昆山市震川西路 186 号	31.37834	120.9475
180	爱心公园	吴江 318 国道	江苏省苏州市吴江区九东路	31.01145	120.6317
181	沙溪公园	沙溪镇中市街	江苏省苏州市太仓市白云中路 156 号	31.56813	121.0614
182	翡翠湖生态公园	工业园区科营路	江苏省苏州市吴中区科营路	31.37125	120.7357
183	琴湖公园	苏州市常熟市	江苏省苏州市常熟市琴湖路	31.63987	120.7565
184	八坼公园	吴江 227 省道	江苏省苏州市吴江区新源路	31.07327	120.666
185	蒹葭公园	陆家镇联谊路	江苏省苏州市昆山市联谊路	31.31411	121.0495
186	苏苑公园	江苏苏州人民南路苏苑新村住宅区内	江苏省苏州市吴中区苏苑街	31.27572	120.6285
187	吴江市震泽新申农庄	三一八国道路与齐心路交叉口附近	江苏省苏州市吴江区 G318(頔塘路)	30.94102	120.5303
188	莺湖园	吴江晨莺路 8 号	江苏省苏州市吴江区晨莺路 6 号	30.97452	120.6354
189	小茅山公园	苏州市虎丘区	江苏省苏州市虎丘区 Y251(锦峰路)	31.32587	120.4329
190	金山公园	明月湾别墅北门正对面	江苏省苏州市吴中区金山路 215 号-4 幢	31.27414	120.5161
191	石路小公园	金阊区石路渡僧桥堍	江苏省苏州市姑苏区山塘街 2	31.31712	120.5992
192	古樟植物园	金庭镇西山风景区（近堡村环山公路）	江苏省苏州市吴中区 X206(梅园路)	31.12605	120.2967
193	生活艺术法国公园	苏州市吴中区	江苏省苏州市吴中区太湖东路	31.26352	120.6427
194	西城体育公园	国泰南路和小河坝西路交叉口	江苏省苏州市张家港市国泰南路	31.86401	120.5131
195	福园公园	玉山镇寰庆路	江苏省苏州市昆山市环庆路 1588 号	31.4235	120.9267
196	思常公园	玉山镇体育场路	江苏省苏州市昆山市体育场路	31.38319	120.9006
197	沁园春	开发区茂源路与青阳北路交叉口	江苏省苏州市昆山市青阳北路 682 号	31.46454	120.9899
198	南施公园	工业园区南施街(近欧洲城)	江苏省苏州市吴中区方洲支路	31.31374	120.7305
199	西园	其他地区郑和西路 414 号	江苏省苏州市姑苏区西园弄 14 号	31.31575	120.5837
200	樱花园	周市医院对面	江苏省苏州市昆山市迎宾东路 888 号	31.45701	120.9683
201	乐荫园	沙溪镇沙溪古镇	江苏省苏州市太仓市白云中路 168 号	31.56863	121.0614
202	浏岛生态园	其他地区浏太公路	江苏省苏州市太仓市 X202(浏昆线)	31.49543	121.2291
203	飘香园	沙家浜镇沙家浜镇	江苏省苏州市常熟市金桩路	31.549	120.8507
204	钓鱼渚公园(环湖南路)	其他地区环湖南路	江苏省苏州市常熟市环湖南路	31.63544	120.6911
205	五彩园	金阊区国土局旁	江苏省苏州市姑苏区玻纤路 9 号	31.32437	120.5847

206	创元公园	协和门诊部斜对面	江苏省苏州市姑苏区干将西路 878 号	31.30609	120.5897
207	红枫园	旺盛路鑫科电子附近	江苏省苏州市相城区陈塂路	31.40377	120.5592
208	塘湾公园	东吴南路长桥以南	江苏省苏州市吴中区东吴南路 337 号	31.261	120.6243
209	南湖苑公园	沙洞路附近	江苏省苏州市常熟市南湖荡路	31.60075	120.6667
210	康佳花园内部公园	高新区林枫路 5 号	江苏省苏州市虎丘区林枫路 5 号	31.31835	120.5325
211	香溢园	采莲路与春申湖中路交叉口	江苏省苏州市相城区春申湖中路 352 号	31.38067	120.6224
212	清凉园	笠泽路 114 号附近	江苏省苏州市吴江区笠泽路 116 号	31.1516	120.6453
213	糖果世界 2 大 1 小亲子套票	江苏省苏州市新区玉山路 162 号苏州乐园水上世界商业广场 A 区	江苏省苏州市虎丘区玉山路 162 号	31.28737	120.5393
214	娄苑公园	玉山镇娄苑路与紫竹路交叉口东	江苏省苏州市昆山市娄苑路 475 号-b	31.39752	120.9562
215	法制主题公园	苏州市吴中区	江苏省苏州市吴中区葑谊街 25 号	31.28996	120.6558
216	中国藏獒观赏园	苏州市吴中区金庭镇缥缈路太湖西山国家森林公园缥缈峰景区内	江苏省苏州市吴中区飘渺路	31.14169	120.2576
217	桥苑	沿沪大道桥苑(东门)附近	江苏省苏州市昆山市 C258	31.33323	121.0987
218	农民公园	黄垆农贸市场旁	江苏省苏州市吴中区木东线	31.13986	120.4849
219	金姬墩公园	工业园区金鸡湖花园东门对面	江苏省苏州市吴中区环洲路	31.30964	120.715
220	西塘小游园	苏州市吴中区	江苏省苏州市吴中区宝带西路 116 号	31.26947	120.6196
221	独墅湖公园自行车租赁点	苏州市吴中区	江苏省苏州市吴中区万寿街	31.25532	120.7152
222	凝翠园	苏州市金阊区	江苏省苏州市姑苏区虎丘路 2 号	31.32061	120.584
223	古里文化公园	苏州市常熟市	江苏省苏州市常熟市银河路	31.63792	120.8291
224	峰园公园	玉山镇望山南路与包家桥路交叉口附近	江苏省苏州市昆山市水秀路 2188 号	31.43815	120.9377
225	昆山科技教育园区中心湖公园	玉山镇苇城中路与萧林西路交叉口北	江苏省苏州市昆山市 Y004(萧林西路)	31.41148	120.8942
226	湖亭公园	苏州市昆山市	江苏省苏州市昆山市湖亭路 102 号	31.45374	120.8726
227	恩钿月季公园	其他地区新港公路协心路口向南	江苏省苏州市太仓市 X203(浏双线)	31.5483	121.1653
228	桂花公园	澄阳路与富元路交界处附近	江苏省苏州市姑苏区竹辉桥	31.29484	120.6396
229	田园	橘子国花园西侧	江苏省苏州市姑苏区江月路	31.33987	120.5948
230	苏安公园	工业园区苏安新村(苏安幼儿园旁)	江苏省苏州市吴中区苏安路 118 号	31.33331	120.6434
231	绿馨园	三川营造旁	江苏省苏州市姑苏区广济北路	31.34105	120.6011
232	银塘公园	其他地区人民路	江苏省苏州市常熟市天字路	31.71746	120.8737
233	天狮公园	苏州市虎丘区	江苏省苏州市虎丘区狮山路 130 号	31.29608	120.5568
234	黄桥梅花园	苏埭路与太阳路交叉口附近	江苏省苏州市相城区河南村路	31.40422	120.5692
235	滨江公园(昆太路)	昆太路 744 号	江苏省苏州市昆山市昆太路 744 号	31.39268	120.9635
236	桃花源	苏州市吴江市	江苏省苏州市吴中区观音山路	31.27885	120.507
237	玉兰苑	工业园区里河新村内	江苏省苏州市昆山市 X203	31.30404	121.1307
238	健康主题公园	江苏省苏州市常熟市泰山南路	江苏省苏州市常熟市泰山南路 78-4 号	31.66355	120.7626
239	平江新城体育公园	平海路与广济北路交界处	江苏省苏州市姑苏区广济北路	31.34737	120.5992
240	菱湖渚公园	临湖镇环太湖路	江苏省苏州市吴中区 X204(湖滨路)	31.13346	120.4304
241	友谊公园	长江中路与朝阳中路交叉口	江苏省苏州市昆山市朝阳中路 310 号	31.37093	120.9741
242	合丰公园	苏州市昆山市	江苏省苏州市昆山市春阳路	31.34281	120.9989
243	水秀园	水秀路 1405 号	江苏省苏州市昆山市水秀路 1405 号	31.42501	120.9397
244	龙西园	环保局旁	江苏省苏州市吴中区龙西路 256	31.27113	120.6138
245	苏州大学百草园	环香山路瑞园旁	江苏省苏州市吴中区 Y500(环香山路)	31.24971	120.4211

246	锦绣公园	盛希路锦绣广场内	江苏省苏州市昆山市盛希路 28 号	31.35846	121.0242
247	梅花草堂	苏州市太仓市	江苏省苏州市太仓市郑和南路 65 号	31.50387	121.2635
248	常福园	其他地区长江西路	江苏省苏州市常熟市长江路	31.67056	120.7297
249	暨阳湖湿地	苏州市张家港市	江苏省苏州市张家港市 S340(南二环路)	31.84994	120.5154
250	香樟园	苏州市虎丘区	江苏省苏州市吴中区梧桐街	31.21837	120.61
251	吴淞江源	苏州市吴中区	江苏省苏州市吴中区共耀路	31.3017	120.7511
252	李桥园	虞山镇海虞北路与淮河路交汇处	江苏省苏州市常熟市海虞北路 58 号	31.67546	120.7514
253	东渡苑景色公园	江苏省苏州张家港市鹿苑镇东郊古黄泗浦畔	江苏省苏州市张家港市 S338	31.86087	120.6239
254	少卿苑	千灯镇千灯镇千灯景区附近(千灯镇政府东)	江苏省苏州市昆山市秦峰中路 220	31.27028	121.0008
255	暖心园	金阊区南新路 62 号	江苏省苏州市姑苏区南新路 62 号	31.31451	120.6021
256	烟雨园公园	其他地区山湖路 1 号	江苏省苏州市常熟市甸新路	31.64606	120.7236
257	未来公园	暨阳东路与东苑路交汇处	江苏省苏州市张家港市暨阳东路	31.86601	120.5654
258	智林园	其他地区滨河大道	江苏省苏州市常熟市 Y315(民富路)	31.66709	120.9509
259	七姬园	平江区前庙巷 1 号	江苏省苏州市姑苏区前庙巷 1 号	31.32284	120.6211
260	四季园	城厢镇钱家祠弄	江苏省苏州市太仓市县府西路 6 号-7	31.44968	121.1008

Table S2. Fully-adjusted[a] ORs (95% CIs) of targeted outcomes associated with quartiles of distance from a park, stratified by gender

		1st quartile (<600m)	2nd quartile (600-903m)	3rd quartile (903m-1348m)	4th quartile (>1348m)
Asthma	Male	Reference	1.02 (0.71 , 1.48)	1.03 (0.71 , 1.51)	0.76 (0.50 , 1.14)
	Female	Reference	1.03 (0.67 , 1.59)	0.82 (0.53 , 1.28)	0.62 (0.38 , 1.01)
Pneumonia	Male	Reference	0.87 (0.65 , 1.17)	0.81 (0.60 , 1.10)	0.93 (0.68 , 1.26)
	Female	Reference	1.17 (0.86 , 1.58)	1.02 (0.75 , 1.38)	0.90 (0.65 , 1.25)
Rhinitis	Male	Reference	1.09 (0.80 , 1.47)	1.12 (0.82 , 1.52)	0.93 (0.67 , 1.29)
	Female	Reference	1.26 (0.90 , 1.75)	1.11 (0.80 , 1.54)	0.99 (0.69 , 1.41)
Eczema	Male	Reference	0.92 (0.68 , 1.24)	0.82 (0.60 , 1.12)	0.68 (0.49 , 0.96)*
	Female	Reference	1.06 (0.76 , 1.46)	1.03 (0.75 , 1.43)	1.07 (0.76 , 1.50)

[a] adjusted for child's age and sex, environmental tobacco smoking at home, parental education, and parental history of asthma

Table S3. Fully-adjusted[a] ORs (95% CIs) of targeted outcomes associated with quartiles of distance from a park, stratified by father education

	1st quartile (<600m)	2nd quartile (600-903m)	3rd quartile (903m-1348m)	4th quartile (>1348m)

Asthma	College and above	Reference	0.96 (0.68 , 1.36)	0.94 (0.65 , 1.36)	0.67 (0.44 , 1.01)
	High School and below	Reference	1.18 (0.71 , 1.95)	0.98 (0.59 , 1.61)	0.75 (0.44 , 1.27)
Pneumonia	College and above	Reference	0.99 (0.76 , 1.29)	0.92 (0.70 , 1.22)	0.96 (0.71 , 1.30)
	High School and below	Reference	1.04 (0.73 , 1.47)	0.89 (0.63 , 1.24)	0.82 (0.59 , 1.16)
Rhinitis	College and above	Reference	1.07 (0.81 , 1.40)	1.00 (0.75 , 1.33)	0.80 (0.57 , 1.11)
	High School and below	Reference	1.44 (0.98 , 2.11)	1.36 (0.93 , 1.97)	1.16 (0.79 , 1.70)
Eczema	College and above	Reference	1.06 (0.81 , 1.39)	0.88 (0.66 , 1.18)	0.85 (0.62 , 1.16)
	High school and below	Reference	0.90 (0.62 , 1.31)	0.97 (0.67 , 1.39)	0.79 (0.54 , 1.15)

[a] adjusted for child's age and sex, environmental tobacco smoking at home, parental education, and parental history of asthma

Table S4. Fully-adjusted[a] ORs (95% CIs) of targeted outcomes associated with quartiles of NDVI values

	1st quartile	2nd quartile	3rd quartile	4th quartile
Asthma	Reference	1.04 (0.76 , 1.41)	1.09 (0.80 , 1.47)	1.11 (0.82 , 1.52)
Pneumonia	Reference	0.93 (0.75 , 1.15)	0.88 (0.71 , 1.09)	0.97 (0.77 , 1.21)
Rhinitis	Reference	0.94 (0.75 , 1.17)	0.78 (0.62 , 0.98)	0.87 (0.69 , 1.10)
Eczema	Reference	0.98 (0.78 , 1.23)	0.97 (0.77 , 1.21)	1.02 (0.81 , 1.30)

[a] adjusted for child's age and sex, environmental tobacco smoking at home, parental education, and parental history of asthma

Table S5. Testing confounding effect of PM2.5, pet ownership, dampness and mold by including them in the full model and compare the odds ratios with the original full model

	original	+PM2.5	+pet	+dampness	+mold
Asthma	0.70 (0.50 , 0.96)*	0.70 (0.50 , 0.96)*	0.69 (0.50 , 0.95)*	0.70 (0.51 , 0.98)*	0.70 (0.51 , 0.97)*
Pneumonia	0.92 (0.74 , 1.15)	0.92 (0.73 , 1.15)	0.92 (0.74 , 1.16)	0.93 (0.74 , 1.17)	0.93 (0.74 , 1.17)
Rhinitis	0.97 (0.76 , 1.24)	0.95 (0.75 , 1.21)	0.96 (0.75 , 1.22)	0.98 (0.76 , 1.25)	1.01 (0.79 , 1.29)
Eczema	0.86 (0.68 , 1.10)	0.85 (0.67 , 1.08)	0.85 (0.67 , 1.08)	0.87 (0.68 , 1.10)	0.87 (0.68 , 1.10)

The original model is the fully adjusted ORs of the targeted outcomes for the people living farthest from parks compared to people living closest to parks, with detailed results presented in Table 4 in the main text.

Table S6. Correlations between air pollutant concentrations (including PM10, PM2.5, NO2, SO2, O3 and CO) and average NDVI within different buffers (100m, 200m, 500m, 1000m)

	PM10	PM2.5	NO2	SO2	O3	CO

NDVI (100m buffer)	0.27	-0.16	0.03	0.1	0.26	0.13
NDVI (200m buffer)	0.22	-0.1	0.01	0.11	0.2	0.12
NDVI (500m buffer)	0.2	-0.09	-0.02	0.12	0.18	0.13
NDVI (1000m buffer)	0.16	-0.04	-0.04	0.12	0.13	0.14
